# Loop electrosurgical excision procedure (LEEP) under local anesthesia: a retrospective analysis of 435 subsequent cases under a quality assurance program

**DOI:** 10.1007/s00404-025-08060-0

**Published:** 2025-05-23

**Authors:** Ulrike Binder, Alice Wenzl, Judith Lafleur, Clemens Tempfer, Lukas Hefler

**Affiliations:** 1 Karl Landsteiner Institute of Gynaecological Surgery and Oncology, Schultestrasse 1, 4020 Linz, Austria; 2 Ordensklinikum Linz and Konventhospital Barmherzige Brueder, Seilerstaette 2-4, Linz, Austria; 3 Department of Obstetrics & Gynaecology, Ruhr-Universität Bochum, Hoelkeskampring 40, Herne, Germany

**Keywords:** Cervical dysplasia, Analgesia, Pain, Complications, Patients’ satisfaction

## Abstract

**Purpose:**

The aim of this study is to analyze a quality assurance program regarding the switch from loop electrosurgical excision procedure (LEEP) in general anesthesia (GA) to local anesthesia (LA) regarding patients’ perioperative pain levels, patients´ satisfaction, as well as resection margins.

**Methods:**

We performed a single-center retrospective analysis of our quality assurance program including all patients undergoing LEEP at the Department of Obstetrics and Gynecology, Ordensklinikum Linz and Konventhospital Barmherzige Brueder Linz, from January 2021 to June 2024. 435 patients were questioned postoperatively after being treated with LEEP in LA regarding the perioperative pain as measured by a numeric rating scale (NRS) and patients’ satisfaction. Clinical data were collected from the patients’ electronic chart to investigate further parameters.

**Results:**

Mean perioperative pain levels were stated as 1.3 (1.9). Five perioperative complications were reported. In seven out of 435 cases (1.6%) an inpatient readmission or a revision surgery under GA had to be performed. A R0 resection rate of 81.4% could be achieved. In 9.7% and 8.3% a R1 resection rate was reported on the ectocervix and on the endocervix, respectively. 95.3% of patients would choose LEEP in LA as their preferred method of anesthesia for a possible subsequent surgical LEEP procedure.

**Conclusions:**

Our study showed that the implementation of LEEP in LA was successful at our department as a new standard of care, resulting in a high R0 resection status and a high level of patients’ satisfaction. We encourage physicians and organizations to switch from GA to LA when performing LEEP for cervical dysplasia.

## What does this study add to the clinical work

**Table:** 

Despite being performed as a standard clinical procedure in many countries few data are available regarding the change in the surgical approach from performing LEEP in general anesthesia to local anesthesia (LA). Our data show this change is safe and is associated with high short-term satisfaction rates and low pain scores for patients undergoing LEEP in LA.	

## Introduction

Despite the availability of Human Papilloma Virus (HPV) vaccines, cervical intraepithelial neoplasia (CIN) is the most common pre-malignant lesion in women worldwide [1]. Thus, the loop electrosurgical excision procedure (LEEP) is a frequently performed surgical procedure in the field of gynecology for treating high-grade CIN and early invasive cancers [2]. Apparently missing exact numbers it is estimated that in Germany between 90,000 and 150,000 LEEP procedures are performed annually, as well as 6000 procedures in Austria [3–5].

Quality parameters for performing LEEP have been outlined in the 2020 S3 guideline “Prevention of cervical cancer”, endorsed by the German (DGGG) and Austrian (OEGGG) Societies of Gynecology and Obstetrics. These quality parameters include a free resection margin (R0) rate of at least 80%, and intra- and postoperative bleeding complications in less than 3% of LEEP procedures [6].

Regarding the optimal anesthesia management, some academic societies provide specific guidelines for the preferred method of anesthesia, whereas in other countries recommendations have not been specified. The aforementioned 2020 S3 guideline does not recommend a specific anesthesia method when performing LEEP procedures, whereas the UK National Health Service (NHS) mandates that the treatment should be offered in local anesthesia (LA) in at least 80% of cases with a target of 90% appearing attainable [6–8].

Reflecting these differing recommendations LEEP is typically performed under LA in countries such as the US and UK, while in continental Europe, especially in Germany and Austria, LEEP under general anesthesia (GA) is accepted as the standard approach [9]. Clearly, advantages of LA include shorter hospital stays, faster appointment scheduling, and lower LEEP-associated costs [7, 10]. It has also been shown that LEEP in LA results in no difference regarding cone fragmentation, duration of treatment, complications, and the rate of CIN at the resection margin (R1), while resulting in higher patient satisfaction after 14 days and smaller cone volume in comparison to surgery in GA [10, 11]. Another study showed that performing LEEP under LA is safe and results in favorable histological and cytological long-term results [12].

Interestingly, until recently there were no prospective randomized studies comparing GA and LA during LEEP. A randomized trial of 208 patient undergoing LEEP either with LA or under GA demonstrated that LEEP under LA is equally well tolerated and offers patient-reported, as well as procedure-related benefits, over GA. These data support the practice of using LA for LEEP procedures as the standard of care [10].

Based on these data, we implemented a strategy to change our surgical approach from LEEP in GA to LA under close monitoring using a quality assurance program. Data on perioperative parameters such as pain levels, complications, histological outcomes, and patient-reported outcomes were collected. In the present study, we report on a retrospective analysis of our quality assurance program reporting—to the best of our knowledge—the largest series of LEEP in LA published in literature so far.

## Methods

### Patients

We performed a single-center retrospective analysis of our quality assurance program including all patients undergoing LEEP at the Department of Obstetrics and Gynecology, Ordensklinikum Linz and Konventhospital Barmherzige Brueder Linz from January 2021 to June 2024. Approval for this study was obtained by the Institutional Review Board of the Medical University Linz (Johannes Kepler Universität, JKU) (EK 1345/2024). The study was performed in accordance with the Declaration of Helsinki.

Patients were examined in our dysplasia clinic and offered LEEP in LA for the presence of CIN or early invasive cancer. Patients opting for GA were not included in the present analysis. Colposcopy was performed in all patients prior to LEEP. Exclusion criteria included pregnancy, concomitant oncological disease, a known hematologic disorder, and the use of blood-thinning medication. The transformation zone (TZ) was assessed (type 1: TZ fully visible, type 2: TZ partly visible, type 3: TZ not visible) and the respective resection type was set.

LEEP was performed under LA in an outpatient setting. Vasoconstrictive agents were not used. First, a self-retaining, transparent, plastic speculum was inserted into the vagina and the cervix was visualized and disinfected. The cervix was sprayed with xylocaine 10% and then injected with xylocain 1% in all four cervical quadrants, at 3, 6, 9, and 12 o´clock, 1 ml in 10 mm depth and 1 ml 1 mm in the subepithelial tissue. One minute was allowed for the respective local anesthetic agent to provide adequate anesthesia. Then the LEEP was carried out: the cervix as well as the squamocolumnar junction were visualized and an electrical loop with an adequate size in relation to the cervix was used to perform the LEEP. If an endocervical lesion was suspected based on preoperative colposcopy and preoperative biopsy findings, an endocervical excision was performed. This step was performed at the surgeon´s discretion. Furthermore, an endocervical curettage was performed. Hemostasis was obtained with a ball electrode, using the spray coagulation mode.

The primary endpoint of the study was the success rate in terms of completion of the procedure in LA, postoperative readmission rate within 14 days, patients´ satisfaction with LEEP in LA, perioperative pain levels as measured by a numerical rating scale (NRS) and resection margins (R1 vs R0).

Secondary endpoints were the respective correlations between the cone volumes, the R1-resection rate, the need for repeat surgery, the patient´s age, the TZ of the cervix, the patient´s vaginal parity, training level of the surgeon, as well as the number of LEEP procedures in LA performed by each individual surgeon.

Board-certified pathologists examined all LEEP specimens. Dimensions of the LEEP specimen (width, length, height) were reported by the pathologists and a LEEP specimen volume was calculated using the following formula: (1/2) × (4/3) × *π* × (length/2) × (width/2) × depth [13].

Patients were discharged immediately after the LEEP procedure. No specific follow-up examinations were planned. Patients were told to call or visit our emergency gynecological department should any serious medical problems occur. Intraoperative and postoperative complications were noted if they occurred within 14 days after LEEP, i.e., postoperative bleeding, cervical infection, uterine infection, or urinary tract infection. The surgeons were aware of a patient’s wish for future pregnancy and the patients’ reproductive history, and attitude towards future pregnancies, all of which were noted in the patients´ electronic chart, which was readily available at all times.

### Statistics

Patient-reported outcomes like the mean perioperative pain levels were recorded after surgery using a numeric rating scale (NRS) [14]. After completion of LEEP, patients were asked if they would choose the LEEP in LA again for possible subsequent LEEP procedures.

The study data were processed in a Database using Microsoft Excel and prepared for statistical analyses using SPSS **(**SPSS 26.0, SPSS Inc., Chicago, IL). Descriptive statistics reporting means (standard deviations), medians (ranges) were reported after normality was checked using Kolmogorov–Smirnov tests. As no upfront sample calculation was conducted, a *p*-value < 0.05 was interpreted descriptively, showing statistical trends or tendencies. In three multivariate models, the dependent variables cone volume in cubic millimeters, the R1 resection rate as a dichotomous variable, and the need for inpatient readmission or a revision surgery under GA as dichotomous variable were correlated with the independent variables patients’ age, the type of TZ, the vaginal parity, the level of training of the LEEP surgeon (resident vs. senior physician) and the number of performed LEEP procedures by surgeon in LA (0–5; 6–10; 10–20; more than 20).

## Results

Between January 2021 and June 2024, a total of 435 patients underwent LEEP in LA. Mean patients´ age was 37 (10.6) years, with a range of 19 to 67 years. Indications for LEEP in LA were CIN III, CIN II, and CIN I proven by cervical biopsy prior to LEEP in 236 patients (54.3%), 66 (15.2%), and 27 (6.2%), respectively. Further indications were abnormal cytological PAP smears indicating HSIL in 86 (19.7%) cases. LEEP in LA was performed in 20 (4.6%) patients who had no cervical dysplasia but underwent LEEP because of postcoital bleedings caused by cervical ectopy. In 75.3%, 13.6%, and 11.1% a TZ Type 1, TZ Type 2, and TZ Type 3 were present, respectively. A total of 25 surgeons performed LEEP procedures in LA: 8 residents, 17 senior physicians. Each surgeon performed a mean of 17.4 (24.3) procedures. 8 surgeons performed 0–5, 7 surgeons 6–10, 5 surgeons 11–20, and 5 surgeons performed more than 20 procedures.

**Fig. 1:**
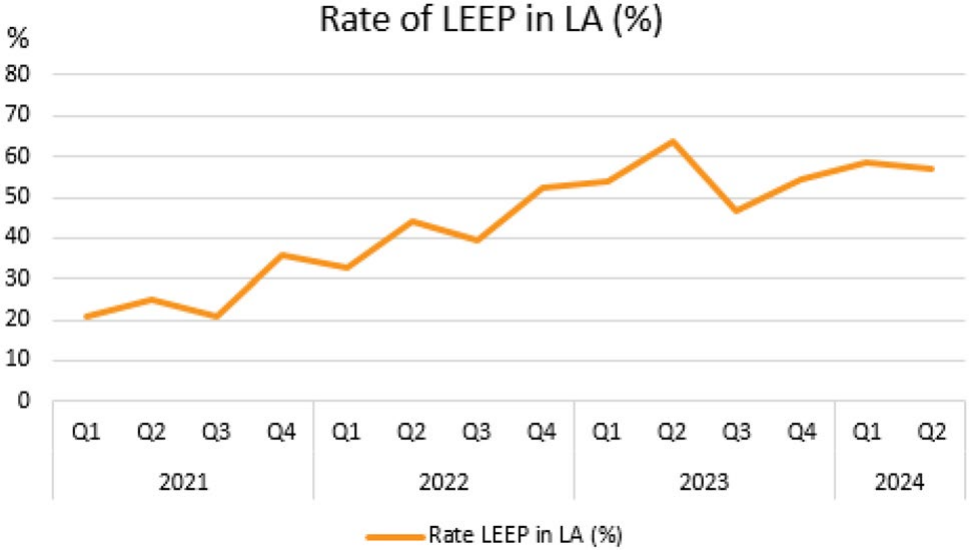
Rates of loop electrosurgical excision procedure (LEEP) (%) in local anesthesia (LA) broken down by the total number of LEEP procedures by quartals (Q) during the quality assurance program

Rates of LEEP (%) in LA broken down by the total number of LEEP procedures by quartals during the quality assurance program are shown in Fig. 1.

Mean perioperative pain levels as measured by an NRS were stated as 1.3 (1.9). In 2 cases (0.5%) a termination of the surgery was necessary; in one case due to a seizure prior to application of the local anesthetic; in one case the LEEP procedure in LA had to be converted to GA due to an injury of the vaginal wall and consecutive bleeding (case no. 21). Five perioperative complications were reported. One patient fainted immediately after LEEP, but recovered without any medical intervention. In two cases an injury of the vaginal wall occurred, which had to be treated with sutures in LA. One patient developed an allergic reaction to the LA, managed through an anti-allergic intravenous therapy. One patient had the primary manifestation of epilepsy prior to administration of any medication.

In seven out of 435 cases (1.6%) an inpatient readmission or a revision surgery under GA had to be performed. An R0 resection rate of 81.4% could be achieved. In 9.7% and 8.3%, an R1 resection rate was reported on the ectocervix and on the endocervix, respectively. After completion of LEEP, 95.3% of patients stated that they would choose the LEEP in LA again. Multivariate models showed that cone volume increased with a higher number of performed LEEP procedures (*p* = 0.001) as well as increasing vaginal parity (*p* < 0.001). A negative correlation could be shown between the TZ and the cone volume (*p* = 0.007). No correlation was found between patients’ age, parity, number of performed LEEP procedures with the R1-resection rate (*p* = 0.3, *p* = 0.4, *p* = 0.3, respectively), and inpatient readmission or a revision surgery under GA (*p* = 0.8, *p* = 0.2, *p* = 0.4, respectively).

No significant correlation could be shown between the level of training and the cone volume, R1-resection rate or the rate of revision surgery under GA (*p* = 0.7, *p* = 0.7, *p* = 0.3, respectively).

## Discussion

Despite its widespread use in many countries worldwide, relatively few data are available on the perioperative outcomes of LEEP in LA and only one randomized study has been published assessing the mode of anesthesia for LEEP [10]. LEEP is still performed in GA in almost all gynaecological departments in Germany and Austria. Implementation of LEEP in LA seemingly poses an obstacle both surgeons, organizations, and patients. Therefore, we report on a large series of LEEP procedures during the implementation phase of LEEP in LA.

Our aim in implementing LEEP in LA was to improve patients’ treatments and outcomes. Although it has been shown that LEEP in LA is also cost-effective obviating the need for anesthesia staff and equipment [9], the reduction of cost or reducing the necessary time of anesthesia personnel was not the focus of this study.

Our present analysis reporting on a large series of LEEP procedures in LA during the implementation phase and the switch from GA to LA at our department has various shortcomings and strengths. Strengths are the relatively high number of subsequent LEEP procedures in LA reflecting “a real-life clinical setting” of a large department of gynaecology in Austria, allowing for the extrapolation of our data to comparable units and departments. Furthermore, we report on all necessary data to evaluate procedural safety and effectiveness as well as readmission rates.

Shortcomings include, firstly, the retrospective analysis of data obtained in the course of a postoperative questionnaire, completed by information from the electronic patient records. Secondly, we did not investigate patients’ or physician’s reasons when opting for GA vs. LA. Published data regarding the patient’s choice for or against LA in different fields of medicine show that the main reasons when opting for GA over LA are concerns about pain during surgery and anxiety regarding the surgical procedure [15]. On the other hand, older and highly extroverted patients seem to be more likely to opt for LA [16]. Thirdly, we cannot provide data on patients’ long-term satisfaction as we did not contact the patients after hospital discharge.

We have observed a steady increase in the rate of LEEP in LA broken down by the total number of LEEP procedures during the time frame of our quality assurance program ranging from 21% up to 64%, depicted in Fig. 1. Of note, there is still room for improvement reaching the goal of 80% as mentioned by the NHS guidelines [8].

Our data are comparable and consistent with previous studies reporting a high patients’ satisfaction rate with adequate safety when treated by LEEP in LA. R0 resection rates in our study were within well-defined standards. Perioperative complication rate was low. We achieved an R0 rate of 81.4% and a readmission rate of 1.6%, staying well within the recommended ranges. Regardless of the surgeon’s level of training the oncological safety was ensured.

Having evaluated our quality assurance program regarding the switch from GA to LA when performing LEEP procedures, we can state that in our hands LEEP in LA is a safe procedure with high patients’ satisfaction. The implementation of LEEP in LA was successful in our department representing a new standard of care. Based on our clinical setting and our data obtained in a “real world” clinical setting we are confident that our data might be extrapolated to various departments or outpatient clinics treating women with cervical dysplasia.

## Data Availability

Data is provided within the manuscript.
